# Climate and ecological constraints of cultivating bioenergy crops for climate mitigation in tropical regions

**DOI:** 10.1093/pnasnexus/pgag123

**Published:** 2026-05-12

**Authors:** Robert Fofrich Navarro, Alcen Chiu, Elsa M Ordway

**Affiliations:** Institute of the Environment and Sustainability, University of California, Los Angeles, Los Angeles, CA 90095, USA; Department of Economics, University of California, Los Angeles, Los Angeles, CA 90095, USA; Institute of the Environment and Sustainability, University of California, Los Angeles, Los Angeles, CA 90095, USA; Department of Ecology and Evolutionary Biology, University of California, Los Angeles, Los Angeles, CA 90095, USA

## Abstract

Negative-emission approaches, such as bioenergy with carbon capture and storage (BECCS), are expected to play a crucial role in mitigating climate change. However, the capacity for biological carbon sequestration under changing climatic conditions remains highly uncertain, particularly in historically warm regions. Although bioenergy can be derived from various biological materials, nearly all operational BECCS facilities capture CO_2_ from bioethanol fermentation. We therefore assess the share of bioenergy crop cultivation that will be exposed to future climate conditions beyond their historically safe climate space (SCS), focusing on tropical regions. We find that 30.5 to 40.3%, 10.4 to 13.0%, and 7.4 to 10.2% of existing oil palm, sugarcane, and soybean production will occur in locations surpassing the SCS of these crops by 2060 under lower warming scenarios. If farmers are unable to adapt effectively to emerging climatic conditions, the carbon sequestration capacity of bioenergy crops in tropical regions could substantially decline under the 2 °C warming target, with reductions of up to 67% for oil palm. However, if farmers instead overcome climatic constraints and expand cultivation in the tropics, conversion of land with an average aboveground carbon storage of around 0.45 Tg C per 100 km^2^ and roughly 600 individual species per 100 km^2^ would result in substantial reductions in ecosystem carbon and biodiversity losses that could outweigh the climate benefits of biofuel-based mitigation.

Significance statement This research highlights the challenges of expanding bioenergy crop cultivation in the tropics, a potentially crucial strategy for offsetting human-driven carbon emissions and mitigating climate change. Our analysis shows that substantial portions of the tropics will become unfavorable for bioenergy crop cultivation due to climate change, precisely when climate models show that increased negative emissions are most needed. If, instead, projected climatic changes are overcome, expanding bioenergy crop cultivation in tropical regions could severely impact natural carbon storage and biodiversity. These findings underscore the need to critically evaluate the ecological costs and long-term viability of bioenergy strategies in tropical regions, while recognizing the complex trade-offs between climate goals and conservation.

## Introduction

Limiting climate warming to ∼2 °C will likely require negative emissions from biotic and abiotic sources (1–6). While abiotic approaches such as direct air capture show promise, they remain prohibitively costly and are not yet scalable to levels sufficient enough to meaningfully offset anthropogenic CO_2_ emissions. In contrast, biological negative-emission approaches, such as bioenergy with carbon capture and storage (BECCS), are more technologically mature, but first-generation biofuel feedstocks require substantial land area (1, 4, 7–9), raising concerns about potential land-use conflicts among negative-emission deployment, ecosystem conservation, and food production (10–13). Climate change further exacerbates these challenges by constraining where feedstocks can be viably cultivated (14–16). Biofuels can be derived from various sources, each of which presents unique environmental, economic, and technological challenges. First-generation biofuels are generally derived directly from food crops and require less processing, but they also put additional pressure on the global agricultural system and can lead to further land clearing and loss of intact ecosystems. In contrast, second- and third-generation biofuels are made from agricultural residues and high-oil-content biomass, such as algae, but require more complex and costly production methods (17–20).

Despite ongoing constraints, continued investments in carbon-intensive technologies suggest that future emission reductions alone may not be sufficient to stabilize temperatures 2 °C. As a result, integrated assessment models often rely on BECCS to account for a substantial portion of negative-emission capacity in lower climate change scenarios (13, 21). However, rapidly scaling first-generation biofuel production could accelerate rates of land-use conversion, which is particularly concerning in regions already experiencing rapid transformation (1, 22–24). Land clearing is pronounced in the tropics, where high demand for agriculture has driven persistently high deforestation rates over the past several decades (25–29). Although rates of agricultural expansion have slowed globally, tropical regions continue to experience substantial land conversion driven by increased agricultural demand, a trend that is expected to persist in the coming decades (25, 30, 31).

While there is a growing body of evidence on the land-use ramifications of biotic negative-emission technologies (5, 13, 32–34), much of this research has focused on land-use conversion, carbon sequestration rates, and potential conflicts with agricultural systems at the global scale (1–4, 35, 36). Yet limited attention has been given to how climate change may modify the climatic conditions experienced by first-generation biofuel feedstock crops in tropical regions. Studies based on large, coordinated modeling efforts, including the Agricultural Model Intercomparison and Improvement Project (AgMIP) and the Global Gridded Crop Model Intercomparison (GGCMI), have used process-based crop models driven to assess future yield responses across major crops and regions (37–40). These studies generally project that climate change will result in yield declines for major crops in the tropics under ∼2 °C of warming but demonstrate substantial spatial heterogeneity in yield responses across tropical regions and crop types. Similarly, the ecological trade-offs associated with expanding bioenergy crop cultivation into tropical ecosystems remain under-evaluated despite the competing demands for land in the region. To address this gap, we assess the climatic and ecological constraints of cultivating first-generation bioenergy crops in the tropics under a 2 °C climate pathway, and also evaluate whether this mitigation strategy remains feasible if global warming exceeds internationally agreed upon targets and instead reach 2.5 °C of warming (i.e. ssp126 and ssp245). We focus on first-generation biofuels due to their feedstock availability, scalability, and ease of integration into the existing energy system, as well as their primary role in current BECCS operations worldwide (17, 41–44). Moreover, first-generation biofuel crops offer an immediately scalable route for rapid BECCS deployment, as existing facilities depend almost entirely on capturing CO_2_ from bioethanol fermentation (42–46). However, it should be noted that our study does not evaluate the adaptive capacity of farmers or future crop cultivars nor does it consider the effects of CO_2_ fertilization on crop cultivation, which might alter the results presented here. Instead, we estimate the share of current bioenergy crop cultivation that occurs in regions where the climate is expected to shift outside historical growing conditions under both the 2 and 2.5 °C scenarios.

## Results

**Figure 1:**
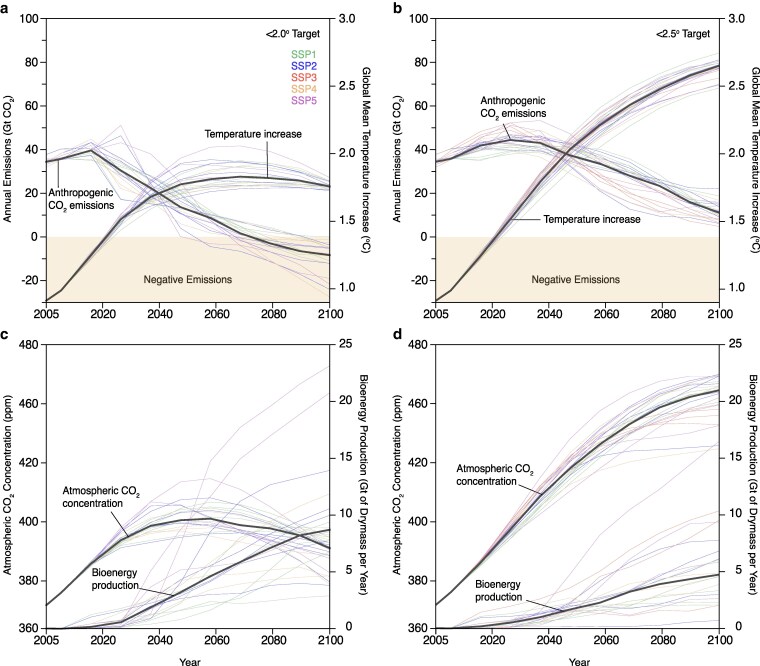
Future temperature, bioenergy production, anthropogenic CO_2_ emissions, and atmospheric CO_2_ concentration projections. a and b display future temperature projections based on SSP data hosted by the IIASA alongside anthropogenic emissions. In contrast, c and d focus on atmospheric CO_2_ concentrations and compare them with bioenergy cultivation. In all cases, the colored lines represent individual SSP ensemble projections, while the black line depicts median projections. a and c represent pathways with a <2 °C increase, while b and d illustrate scenarios to stabilize future climate at <2.5 °C.

In lower warming scenarios, global mean temperature is largely stabilized through a combination of steep anthropogenic emission reductions and a substantial increase in bioenergy production (Fig. 1). Under the 2 °C scenario, annual anthropogenic CO_2_ emissions peak at ∼40 Gt CO_2_ per year before reaching net zero between 2060 and 2080 (Fig. 1a). Similarly, median atmospheric CO_2_ concentrations plateau in the late 2050s and 2060s in the 2 °C scenario, with average scenario bioenergy production increasing to ∼8 (2.5 to 14) Gt of dry biomass per year in 2100 (Fig. 1c). In contrast, in the 2.5 °C scenario, median atmospheric CO_2_ concentrations reach their maximum around the end of the century, and bioenergy production rises to nearly 5 (1 to 16) Gt of dry biomass per year in 2100 (Fig. 1d). Many mitigation pathways that successfully avoid more extreme climate warming assume large-scale deployment of negative-emission technologies, particularly BECCS. However, the median life-cycle cost of BECCS is ∼$200 per ton of CO_2_, which is higher than most nature-based options but lower than the median estimate for direct air capture and storage (Fig. S1 and Table S2). In some cases, BECCS costs exceed $400 per ton of CO_2_ removed, surpassing the costs of other carbon-removal approaches, including carbon capture and storage, enhanced weathering, and soil carbon sequestration, by $50 to $300 per ton of CO_2_ removed.

**Figure 2:**
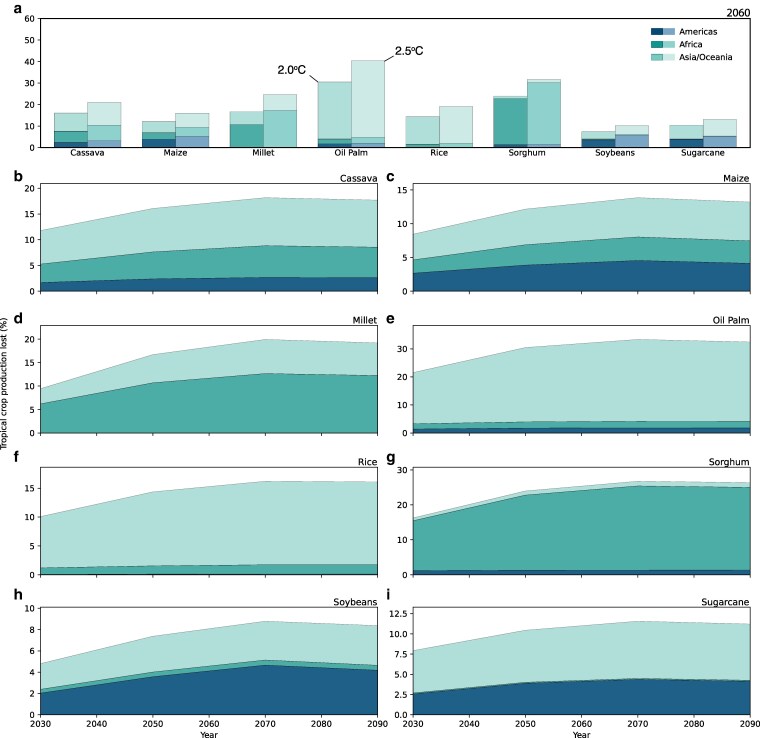
Share of crop production occurring in tropical areas exceeding their SCS under future climate scenarios. a) The proportion of total crop production occurring beyond its historical SCS in 2060 under 2 and 2.5 °C warming, illustrated by region (colors). b–i show how tropical crop production shifts outside its SCS over time under 2 °C of warming.

Despite climate models projecting an increase in global bioenergy production by midcentury, our analysis reveals that a substantial share of current crop production occurs in regions that will be exposed to conditions outside their historical safe climate space (SCS), limiting the availability of feedstocks for biofuel production. Notably, a large share of existing rainfed crop production occurring in the tropics resides in locations that will experience agriculturally unfavorable climates by 2060 under 2 and 2.5 °C of warming. This specifically includes 30.5 to 40.3%, 14.4 to 19.0%, 10.4 to 13.0%, and 7.4 to 10.2% of oil palm, rice, sugarcane, and soybean existing production in the tropics (Fig. 2a). However, there is substantial heterogeneity across tropical regions, as larger shares of oil palm, sorghum, and soybean production in Asia, Africa, and South America, respectively, would be exposed to unfavorable conditions by midcentury. As climate change progresses, there is a nearly consistent increase in the fraction of production exposed to climatic conditions exceeding their SCS through the century (Fig. 2b–i). Notably, at its peak in 2070, over 30% of oil palm cultivation takes place outside its optimal climate range, whereas ∼11% of sugarcane production is expected to face unfavorable climatic conditions in the same timeframe.

**Figure 3:**
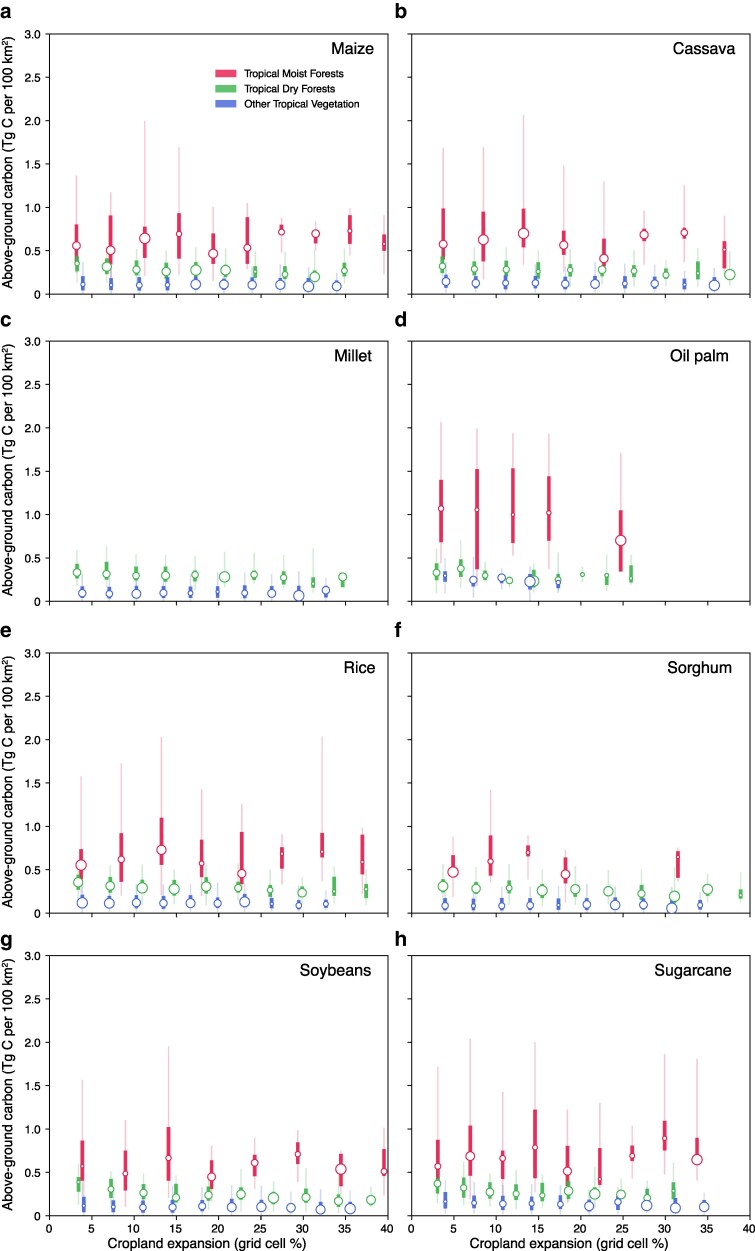
Cropland dynamics in tropical regions. Panels a–h show the relationship between cropland expansion (*x*-axis, % of grid cell) and aboveground carbon storage (*y*-axis, Tg C 100 km^2^) for a given rainfed crop used for bioenergy production. Vertical whiskers indicate the 5th–95th percentile (thin line) and 25th–75th percentile (thick line) of carbon storage in binned cropland expansion intervals, weighted by cropland area within each ecoregion. Colors show tropical moist forests (red), tropical dry forests (green), and other tropical vegetation (blue). White-filled circles with colored edges indicate the weighted mean carbon storage in each bin, with circle size corresponding to the relative production of each crop. Gold and dark green stars mark the weighted-mode and 90th-percentile intersections between crop production and carbon storage, respectively.

**Figure 4:**
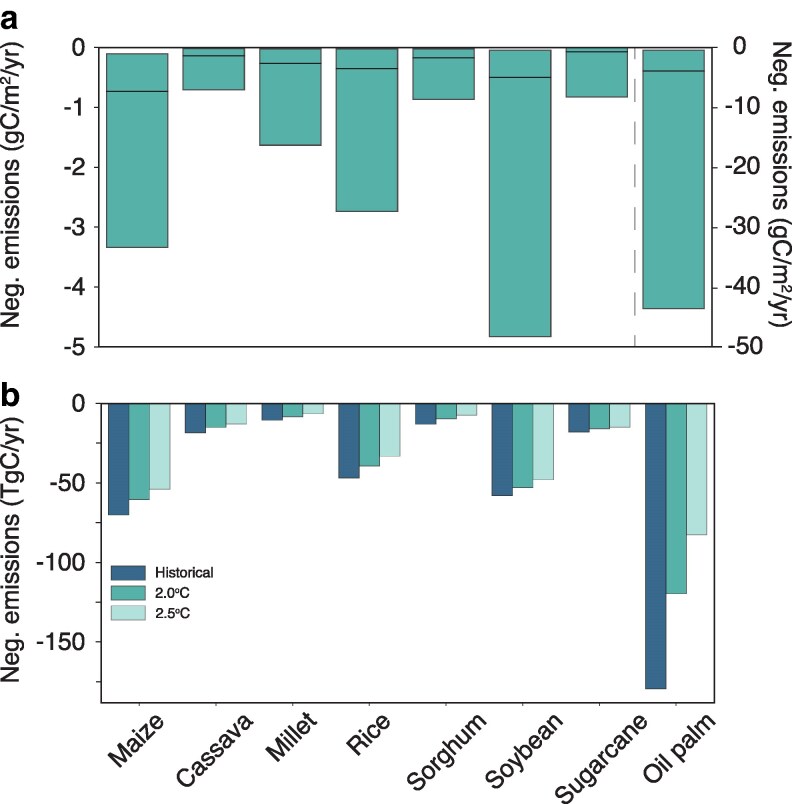
Change in cropland negative-emission potential. a) The grid-cell distribution of negative-emission potential for bioenergy crops (left *y*-axis) and for oil palm (right *y*-axis). b) Projected changes in the negative-emission potential of bioenergy crops under historical conditions and future climate scenarios (colors) aggregated across tropical regions.

Historically, crop production has been highest in locations outside tropical moist forests, in regions where the average annual vegetation carbon density is <0.5 Tg C per 100 km^2^ (Fig. 3). Still, the increased demand for biofuels could incentivize farmers to expand crop cultivation into previously undisturbed areas, posing a direct trade-off with the carbon-sequestration capacity of neighboring ecosystems. Cassava, rice, sorghum, and soybean are the predominant crops that have displaced moist tropical forests and are cultivated in regions where 7.5 to 13% of these forests have been converted to croplands (Fig. 3b, e–g). In dry tropical forests and shrublands, soybeans, sorghum, rice, and maize have been cultivated in areas where, over the past two decades, cropland expansion has displaced 8–10% of these ecosystems. Unsurprisingly, carbon sequestration rates in natural environments generally far exceed those observed in croplands across tropical regions, often by orders of magnitude (Fig. S2). Net primary production rates are highest along the western Amazonian Basin, southeastern Brazil, the Congo Basin, and northern Laos and Myanmar, where annual carbon uptake reaches ∼0.15 Tg C per 100 km^2^ (Fig. S3). Similar levels of carbon uptake are observed in only a few agricultural regions, notably sugarcane and soybean fields in southern Brazil and oil palm plantations on the Malaysian Peninsula and the islands of Borneo and Sumatra (Fig. S3k, t, and w). The highest carbon uptake potential for biofuel is produced from oil palm, soybean, and maize (Fig. 4a). However, the ability of these crops to continue sequestering carbon is largely diminished in future climate warming scenarios (Fig. 4b).

**Figure 5:**
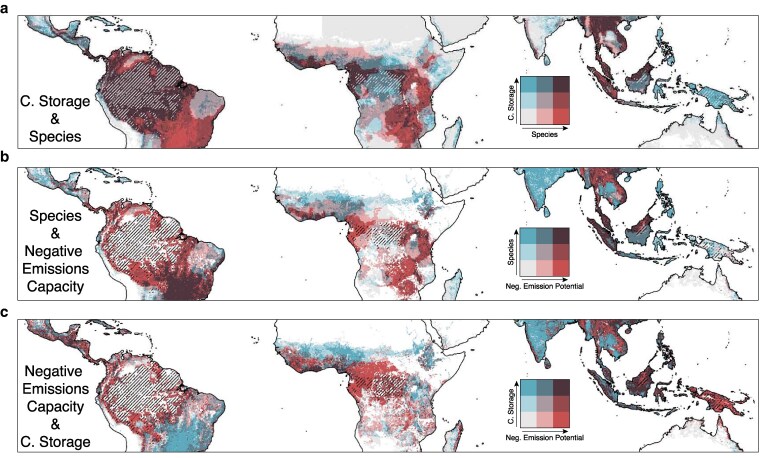
Choropleth bivariate maps of negative-emission potential and ecological trade-offs from expanding biofuel crops in historically cultivated areas. In a, bluer areas indicate a higher concentration of AGB, while red hues suggest greater species richness, and hatches show areas of intact tropical forests. a and c compare species richness and AGB with the average negative-emission potential from crops included in this study. Red hues represent negative-emission potential, while blue colors indicate the trade-offs in these panels. In b and c, white areas indicate regions where none of the crops included in this study have been historically cultivated.

Large trade-offs exist between biofuel crop production, existing carbon sequestration by tropical ecosystems, and biodiversity conservation in tropical regions, particularly if crop management efforts successfully mitigate anticipated climate damages (Fig. 5). On average, tropical regions store ∼0.45 Tg C and host ∼600 individual vertebrate species per 100 km^2^, whereas intact tropical forests contain ∼1.47 Tg C per 100 km^2^. Our analysis also compares the species richness and average annual live biomass carbon storage across tropical ecosystems, revealing that expanding crop cultivation for biofuel production could have adverse effects on biodiversity and natural carbon storage across the tropics. We find more favorable conditions for biofuel crop cultivation in Central America, southern Mexico, Indonesia, Brunei, Malaysia, and the Philippines. However, expanding crop production in these areas would directly conflict with locations of high biodiversity and carbon storage, as these regions are among the most biodiverse on the planet, with an average of 800 to 1,200 individual species per 100 km^2^. Therefore, expanding crop cultivation within the Amazonian basin, northern Myanmar, Laos, Thailand, Malaysia, Bhutan, the Republic of Congo, Cameroon, Gabon, and Equatorial Guinea could lead to substantial declines in natural carbon storage and biodiversity (Fig. 5a). Conversely, southern Brazil, Malawi, Tanzania, and Zambia show high species richness (e.g. 649 to 829 individual species per 100 km^2^) but low levels of aboveground biomass (AGB; ∼0.12 to 0.27 Tg per 100 km^2^). Much of Papua, the Philippines, eastern Madagascar, parts of eastern India, and the Mexican highlands exhibit relatively lower species richness (∼264 to 554 individual species per 100 km^2^) with moderate AGB (∼0.30 to 0.97 Tg C per 100 km^2^). In addition to carbon stocks and species richness, we also examined the spatial relationship between croplands and remaining intact tropical forests. Many crop areas are directly adjacent to intact forested regions (Figs. 5 and S6), particularly in the Amazon and Congo Basins and across the island of Borneo. These regions contain some of the last extensive, intact tropical forests, and have some of the highest concentrations of species (1127, 583, and 716 individual species per 100 km^2^ area, respectively) and aboveground carbon stocks (0.96, 0.66, and 0.92 Tg C per 100 km^2^, respectively) on the planet. The Amazon alone contains 570,763 km^2^ of intact forest, accounting for 68.4% of all intact tropical forest area, while the Congo Basin and Borneo hold ∼120,896 and 16,560 km^2^, respectively.

## Discussion

Stabilizing global temperatures at 2 °C will likely require negative emissions combined with the rapid phaseout of CO_2_-emitting technologies (3, 4, 47, 48). Immediate and steep CO_2_ emissions reductions may reduce the need for bioenergy, whereas delayed decarbonization would likely increase the need for extensive negative-emission deployments to meet more stringent climate targets (21, 49, 50). First-generation biofuels face substantial challenges in tropical regions, potentially limiting their role in mitigating climate change (18, 19). Even under modest warming scenarios, changing climatic conditions potentially undermine the feasibility of this mitigation pathway if bioenergy deployment is substantially delayed, unless cultivation expands into areas with more favorable conditions to compensate for climate-induced losses (51). Process-based crop model ensembles (e.g. GGCMI and AgMIP) indicate that some major crops may not experience substantial declines in certain regions due to CO_2_ fertilization and increased adaptation efforts despite an additional ∼2 °C of warming (38, 40). Our results do not contradict these findings but rather identify where future conditions depart from the climatic envelope under which current crop production has occurred. It should be noted that exceeding this envelope does not imply immediate yield decline but highlights where farmers will have to adapt to new conditions (e.g. by increasing their dependence on untested cultivars, management, and resource inputs). Still, overcoming future climatic barriers to first-generation bioenergy production would provide minimal mitigation of the broader tension between carbon-removal goals relying on land-intensive strategies and biodiversity preservation across the world's most species- and carbon-rich landscapes.

Rising demand for food and energy is expected to intensify pressure on existing agricultural land, potentially leading to further expansion and substantially reducing net carbon storage because these crops retain only a fraction of the carbon and species present in native vegetation. While the infrastructure needed to scale biofuel production in tropical regions remains underdeveloped and lacks the economic framework to support rapid expansion (52, 53), these limitations may be overcome as future demand for bioenergy grows. To deliver lasting climate-mitigation benefits, CO_2_ absorbed during cultivation must be captured at combustion and durably sequestered in long-term geologic reservoirs, such as depleted oil and gas reservoirs, deep saline aquifers, or unmineable coal seams (54–56). Thus, without strategic and coordinated land-use planning, the infrastructure required to support BECCS (e.g. large-scale pipeline networks and road development) would further fragment tropical ecosystems (57–62).

It is important to note that our study does not encompass all crops that can be used to produce bioenergy, nor is it focused solely on those with the greatest potential under future climate scenarios. Instead, we evaluate eight crops widely cultivated in tropical regions that have historically been used for first-generation biofuel production (63–65), irrespective of their carbon sequestration capacity or abundance as primary feedstocks. We also do not account for life-cycle emissions or the climate-mitigation benefits of displacing fossil energy sources, which, although critical, are beyond the scope of this analysis. Similarly, we exclude potential CO_2_ fertilization benefits given the uncertainty surrounding the yield response from elevated atmospheric CO_2_ under ambient conditions in tropical environments (e.g. nutrient limitations and extreme heat exposure), particularly for crops with different photosynthetic pathways (i.e. C_3_ vs. C_4_) (66–68). Our methodological framework, in contrast, isolates how climate exposure in existing croplands is projected to shift with warming, providing a foundation for evaluating the challenges of cultivating first-generation biofuel feedstocks under future scenarios. In some cases, the negative effects of an unfavorable climate may be partially offset by adopting more resilient crop varieties or expanding irrigation, although the effectiveness of these approaches remains uncertain (69, 70). Alternatively, second-generation biofuel feedstocks, such as Miscanthus, Guinea grass, and Napier grass, offer higher carbon sequestration rates and can often be grown on marginal lands, potentially reducing pressure on more productive agricultural areas (34, 71, 72). Yet bioenergy derived from lignocellulosic biomass such as perennial grasses remains economically unviable without the co-production of other high-value products, currently limiting its scalability (72–74).

Ultimately, avoiding the most severe impacts of climate change requires an immediate and sustained shift away from fossil energy sources (21, 47, 75–77), which remain the dominant source of anthropogenic CO_2_ emissions (78). Negative-emission technologies such as BECCS can be essential to ease the transition away from CO_2_-emitting infrastructure and achieve lower mitigation targets (4, 5, 17, 49). However, agricultural production is also a major source of greenhouse gas emissions and a primary driver of global deforestation and species loss (78–80). Therefore, as climate change mitigation strategies are developed and debated, it is essential to balance these efforts with the protection of ecosystem functions that support biodiversity, carbon storage, and human well-being. Species extinctions are irreversible and can trigger cascading effects across both natural and human systems, underscoring the importance of integrated climate mitigation, energy, agriculture, and conservation policy (81–83). Tropical forests store the highest concentrations of aboveground carbon (59) and biodiversity on Earth and support the livelihoods of over a billion people, making them an essential region for evaluating the environmental trade-offs posed by climate-mitigation strategies that intensify pressures on already vulnerable ecosystems. Ensuring that climate-mitigation efforts do not come at the expense of regional ecosystems is critical for delivering truly sustainable and equitable climate change solutions.

## Methods

We evaluated how future climate change could constrain the cultivation of crops historically used for first-generation biofuels in tropical regions. Our analysis focuses on eight rainfed crops that can be used to produce bioenergy (i.e. maize, cassava, oil palm, millet, rice, sorghum, soybeans, and sugarcane). These crops were selected for their established role as bioenergy feedstocks, their prevalence in tropical regions, and the availability of high-resolution data. We adopted the SCS framework to identify the climatic envelope under which current production of these crops occurs and to evaluate where future climate conditions are projected to exceed this historical range in existing croplands within tropical regions.

To define the SCS for each crop, we characterized baseline climate conditions using monthly mean temperature and total precipitation from WorldClim 2 (84), aggregated these values to annual estimates, and mapped each grid point following the Holdridge Life Zone approach (85, 86). Rainfed cropping data were obtained from the Global Agro-Ecological Zones dataset (GAEZ v3.0), produced by the UN Food and Agriculture Organization (FAO) and International Institute for Applied Systems Analysis (IIASA) (87), which provides global crop production at 5-arcminute spatial resolution. For each crop, we extracted the corresponding annual climate values from the historical baseline period, which represents the 30-year climatology averaged from 1970 to 2000. In alignment with past studies, we identified the climatic envelope that encompasses 95% of total global rainfed production (85, 86). The 95th percentile captures the dominant production areas, while the lowest 5% were excluded to remove marginal regions where cultivation occurs under low-yield conditions. Future climate projections were also obtained from the WorldClim 2 database, which provides bias-corrected and downscaled CMIP6 outputs at the same five arcminute resolution. We used monthly mean projections of maximum temperature, minimum temperature, and precipitation from 14 global climate models under the SSP126 and SSP245 scenarios. Each model output represents the average climate over 20-year periods (2021–2040, 2041–2060, 2061–2080, and 2081–2100). We derived multimodel ensemble averages for each variable and period, which were used to quantify projected changes in climate conditions within tropical cropping areas. A complete list of the global climate models used in this study is available in Table S1.

For each grid cell containing crop production within tropical regions, we computed the annual mean temperature and total precipitation for each future 30-year period and compared these values with the historical SCS envelope. Grid cells where future climate conditions remain within the historical bounds were classified as inside the SCS, while those exceeding either the temperature or precipitation limits were classified as outside the SCS. To quantify potential production losses, we applied the SCS masks to the current distribution of rainfed crop production. For each crop and scenario, we calculated the proportion of the existing tropical cultivation area projected to remain within or move beyond its historical climatic range. This approach provides a consistent and spatially explicit measure of how climate change may alter the geographic extent of suitable growing conditions across major bioenergy crops.

Future projections of CO_2_ emissions, atmospheric CO_2_ concentration, and bioenergy production were obtained from the IIASA shared socioeconomic database. We included all available models under the 2 and 2.5 °C scenarios, calculated the median projection, and presented the full range of results. To evaluate the ecological implications of potential cropland expansion, we assessed vertebrate species richness and AGB across tropical regions and compared their distributions with the carbon sequestration potential of BECCS. Vertebrate species richness was derived from the IUCN Red List raster (version 2025-2), which reports the number of amphibian, bird, mammal, and reptile species within each 10 × 10 km cell. Annual AGB carbon values were obtained from Xu et al. (88), with AGB values averaged from 2000 to 2020. Bioenergy carbon sequestration potential was calculated following the Xu et al. (51) methodology and averaged across all bioenergy crops. Each data layer was rescaled to express its value as a percentage of its maximum value and classified into three equal-frequency categories: low (<25th percentile), medium (25th–75th percentiles), and high (>75th percentile) to facilitate visual interpretation. We used the Intact Forest Landscape dataset from Potapov et al. (2017) (89) to illustrate areas where bioenergy expansion could potentially degrade intact forests. We then generated bivariate maps to depict spatial overlap among ecological metrics, BECCS potential, and intact forest areas, highlighting regions where cropland expansion would present the greatest ecological risks.

To compare the carbon uptake potential of croplands cultivated for bioenergy with that of tropical ecosystems, we analyzed net primary production (NPP) and net ecosystem production across both uncultivated and cultivated lands. Natural vegetation NPP was obtained from the Moderate Resolution Imaging Spectroradiometer, which provides annual estimates from 2001 to 2020. Cropland NPP was estimated using the method described in Xu et al. (51), where NPP is calculated as the product of total production, the fraction of dry biomass, the fraction of biomass in shoots, and the fraction of carbon in dry biomass (51). To isolate the contribution of natural vegetation in mixed-use grid cells, we subtracted cropland NPP from the total NPP for each grid point that included one or more of the crops analyzed in this study. Cost ranges for carbon dioxide removal technologies were obtained from the S&P Global 2023 report (90), which aggregated estimates published by the IEA (91) and IPCC AR6 WG III (92). Carbon dioxide removal (CDR) ranges shown in Fig. S1 and Table S2 represent technology-specific life-cycle capital and operating costs expressed in US dollars per ton of CO_2_ removed.

## Supplementary Material

pgag123_Supplementary_Data

## Data Availability

All data used within this study are publicly available. Data processing and analysis were conducted in MATLAB and Python. The underlying code to produce these results is available at https://github.com/rfofrich/Constraints-of-cultivating-bioenergy-crops-for-climate-mitigation-in-tropical-regions.git.
