# Supplementary material for: Climate and ecological constraints of cultivating bioenergy crops for climate mitigation in tropical regions
Source: PNAS Nexus. 2026 May 12;5(5):pgag123. doi: 10.1093/pnasnexus/pgag123 (PMC13162350; doi:10.1093/pnasnexus/pgag123)
Supplement: pgag123_Supplementary_Data [file pgag123_supplementary_data.pdf]

Climate and ecological constraints of cultivating bioenergy crops for climate mitigation in tropical regions

Robert Fofrich Navarro[1*], Alcen Chiu[2], and Elsa M. Ordway[1,3]

[1] *Institute of the Environment and Sustainability, University of California, Los Angeles, Los Angeles, California, United States of America*

[2] *Department of Economics, University of California, Los Angeles, Los Angeles, California, United States of America*

[3] *Department of Ecology and Evolutionary Biology, University of California, Los Angeles, Los Angeles, California, United States of America*

[*]Corresponding author's email address: robertfofrich@dartmouth.edu

Supplementary Materials:

Supplementary Figures 1 - 6

Supplementary Tables 1 - 2

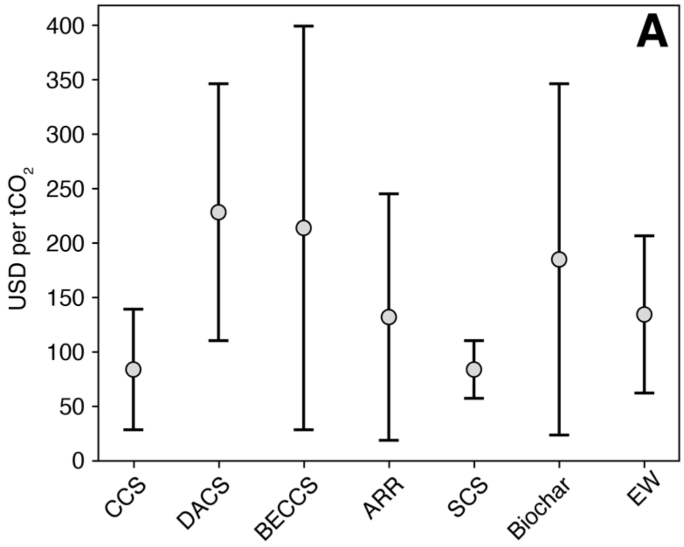

Figure S1 Negative emission technology costs. Atmospheric carbon dioxide removal, also known as negative emissions, is crucial for achieving lower emission targets. However, costs vary depending on the approach and technology used. Median values are indicated by a circle, while the full range is shown by the whiskers. Technologies listed include CCS (Carbon Capture and Storage), DACS (Direct Air Capture and Storage), BECCS (Bioenergy with Carbon Capture and Storage), ARR (Afforestation and Reforestation), SCS (Soil Carbon Sequestration), Biochar, and EW (Enhanced Weathering).

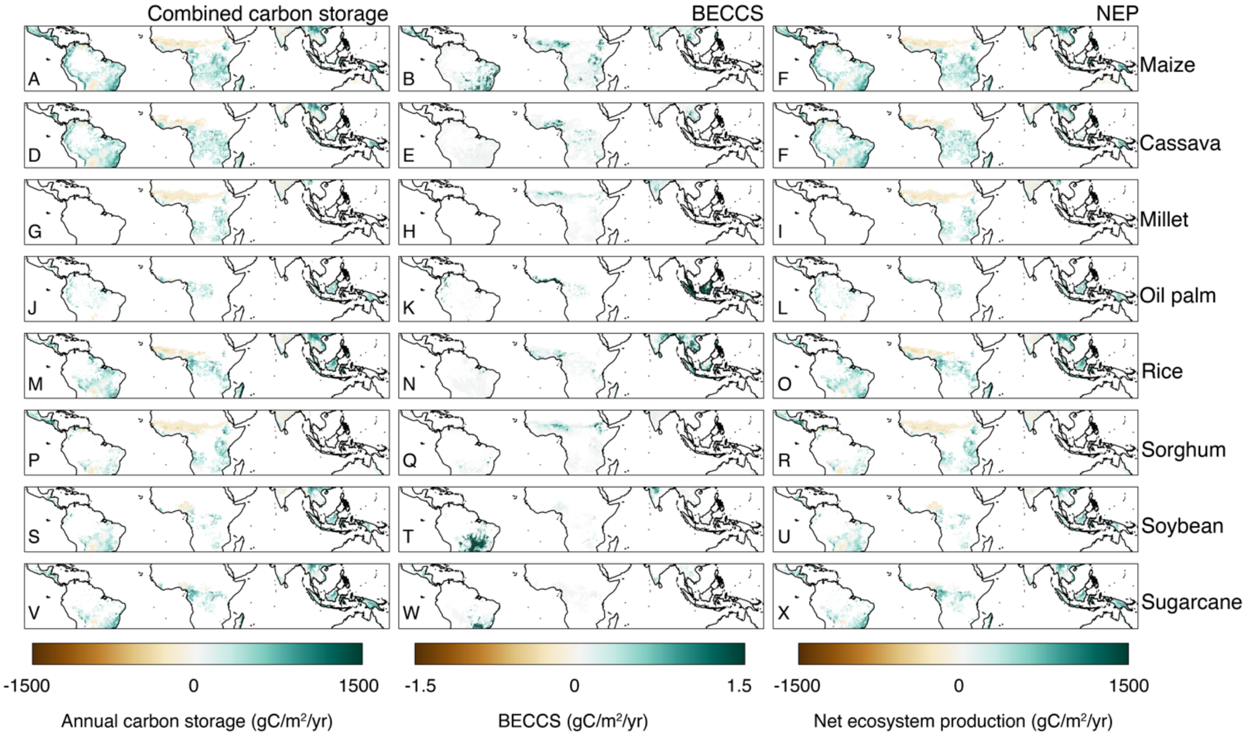

Figure S2 | Carbon storage potential in existing croplands. Maps illustrate the annual carbon storage potential in the natural environment (right column) and contrast this with the carbon uptake by different crops (middle column). In comparison, the leftmost column represents the annual carbon uptake from a combination of BECCS and NEP.

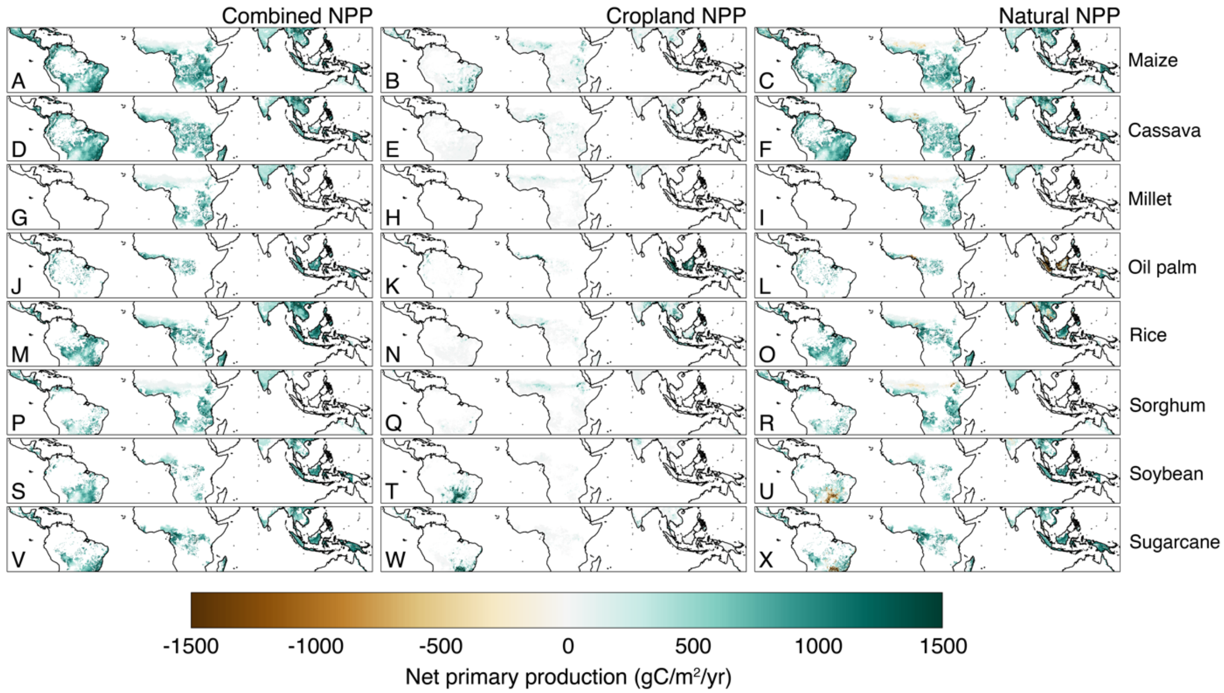

Figure S3 | Net primary production in existing croplands. Maps in the left column highlight the total net primary production from crops and the natural environment. In contrast, the middle panels showcase NPP from crops alone, while the right column highlights the NPP from the natural ecosystem within grid cells that contain cropping areas.

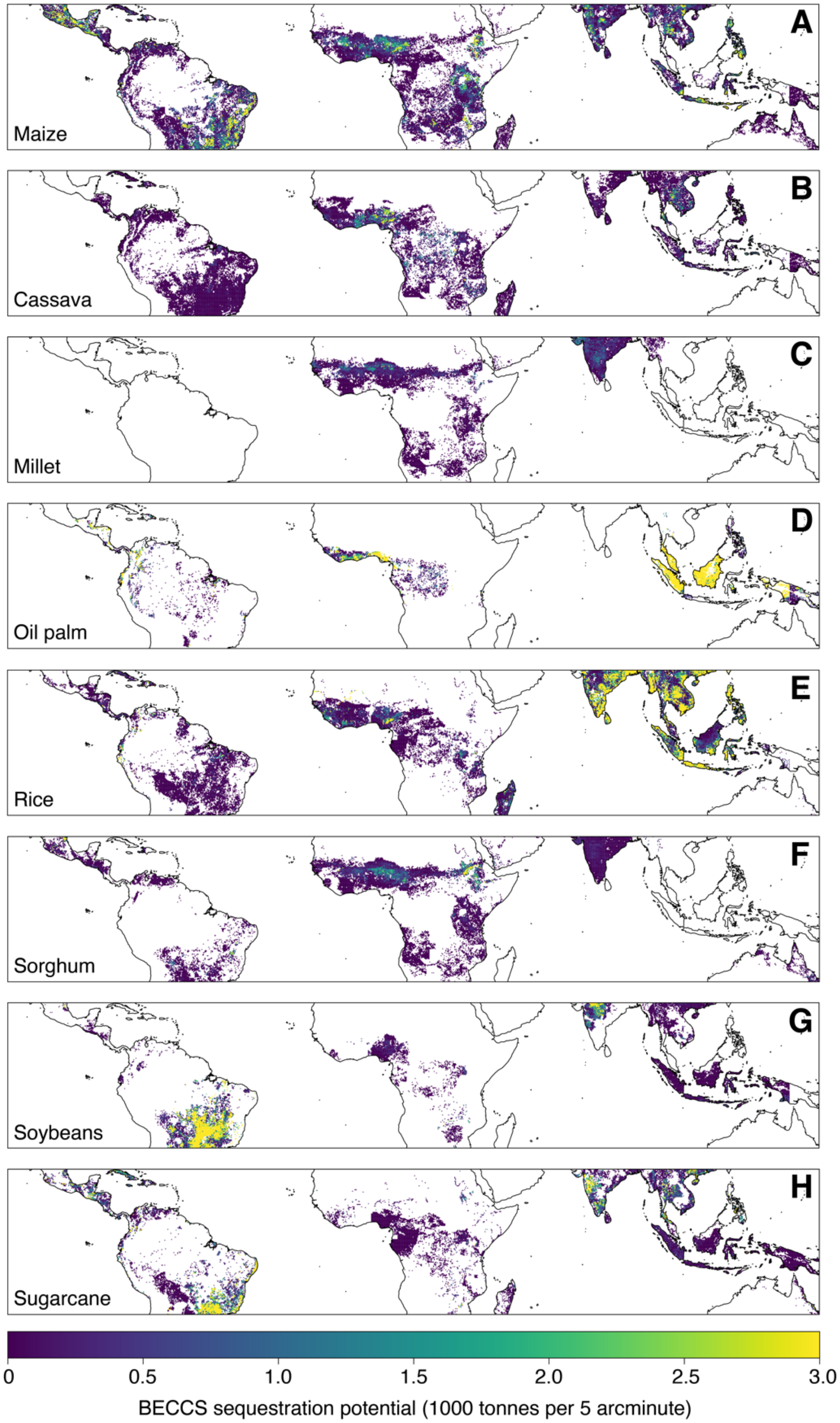

Figure S4 | Maps of crop BECC potential. We highlight BECC sequestration potential in existing croplands based on their historical production levels. Darker colors indicate smaller production values, thus smaller BECC potential, while conversely, brighter colors depict areas where BECC sequestration rates would be greatest.

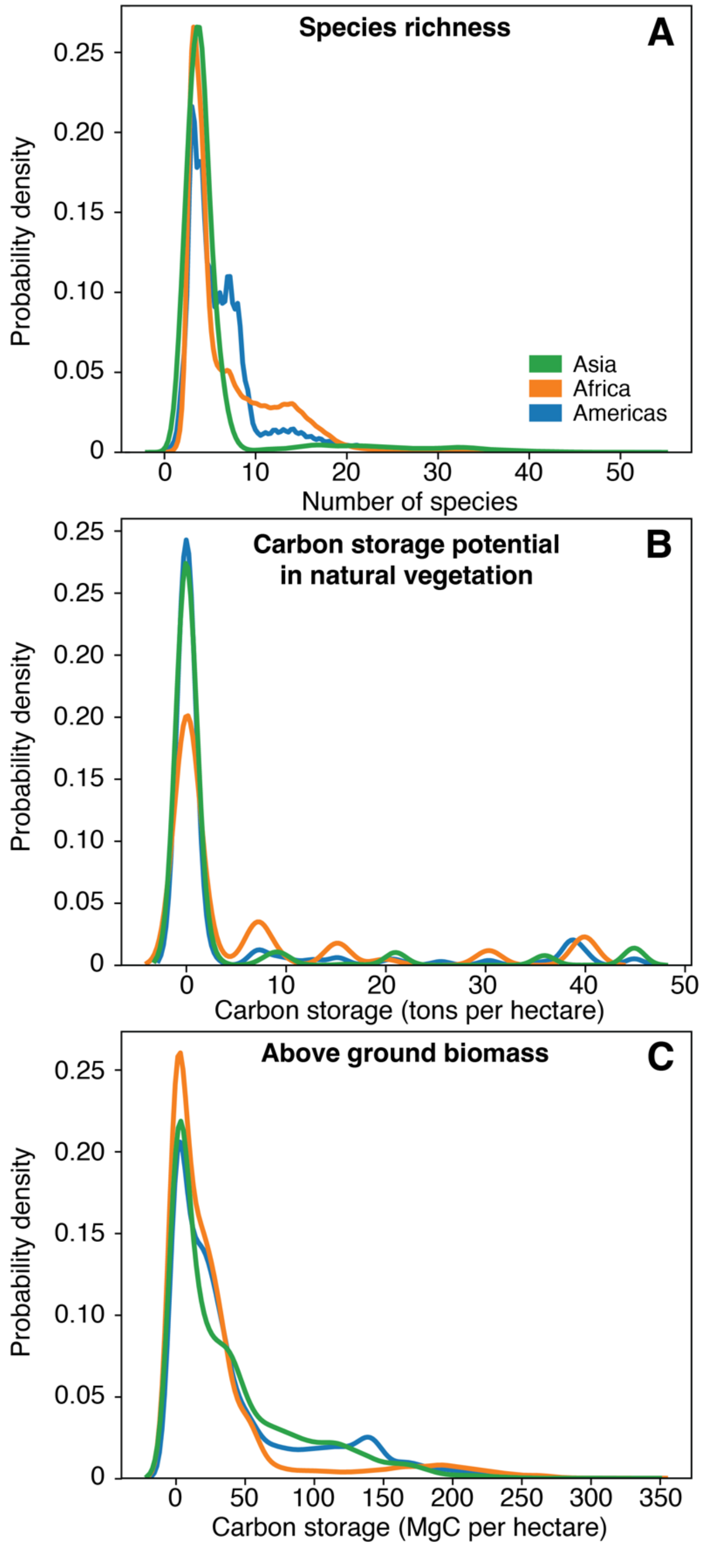

Figure S5 | Tropical carbon storage and species richness distribution. Probability density functions showing the distribution of natural carbon storage, above-ground biomass, and species richness in tropical regions categorized by continent (Asia – green, Africa – orange, and the Americas – blue).

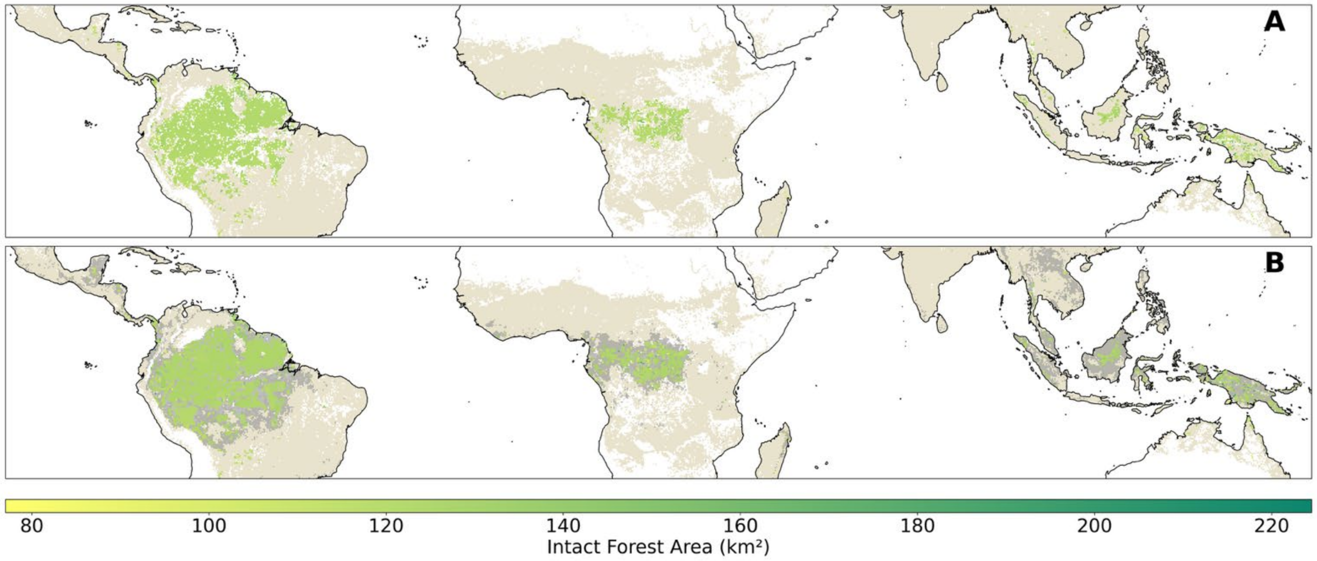

Figure S6 | Tropical Forest extent. The maps highlight regions where remaining intact forests overlap or are adjacent to cropland, underscoring areas at heightened risk of agricultural expansion. Panel A shows intact forest area (colors, million km²) and cropland extent (tan). Panel B illustrates forest fragmentation due to land-use change, with the full forest extent overlaid in gray.

Source name	Institution	Host Country/Region	Equilibrium Climate Sensitivity
**ACCESS-CM2**	CSIRO-ARCCSS	Australia	4.7 C
**BCC-CSM2-MR**	CCCma	Canada	5.6 C
**CMCC-ESM2**	CMCC	Italy	3.6 C
**EC-Earth3-Veg**	EC-Earth-Consortium	EU	4.3 C
**FIO-ESM-2-0**	FIO	China	4.3 C
**GFDL-ESM4**	NOAA-GFDL	United States	2.7 C
**GISS-E2-1-G**	NASA-GISS	United States	2.7 C
**HadGEM3-GC31-LL**	MOHC	United Kingdom	5.6 C
**INM-CM5-0**	INM	Russia	1.9 C
**IPSL-CM6A-LR**	IPSL	France	4.6 C
**MIROC6**	MIROC	Japan	2.6 C
**MPI-ESM1-2-HR**	NCC	Norway	2.5 C
**MRI-ESM2-0**	MRI	Japan	3.2 C
**UKESM1-0-LL**	MOHC	United Kingdom	5.4 C

Table S1 | Underlying global climate models. A total of 11 global climate models were used in this study.

**Negative emission technology**	**Low, median, and high cost estimates in USD per ton of CO_2**		
Carbon Capture and Storage	15	73	130
Direct Air Capture and Storage	100	223	345
Bioenergy with Carbon Capture and Storage	15	208	400
Afforestation and Reforestation	5	123	240
Soil Carbon Sequestration	45	73	100
Biochar	10	178	345
Enhanced Weathering	50	125	200

Table S2 | Global costs of negative emission approaches. Lifecycle cost estimates for negative emission solutions vary by approach and technology. Estimates are in 2023 U.S. dollars per ton of CO_2 and obtained from the S&P Global 2023 report.

Supplemental References

1. S. Santanoo *et al.*, Physiological and Proteomic Responses of Cassava to Short-Term Extreme Cool and Hot Temperature. http://dx.doi.org/10.3390/plants11172307.
2. W. Schlenker, M. J. Roberts, Nonlinear temperature effects indicate severe damages to U.S. crop yields under climate change. *Proceedings of the National Academy of Sciences* **106**, 15594 (2009).
3. N. D. Jackson, M. Konar, P. Debaere, J. Sheffield, Crop-specific exposure to extreme temperature and moisture for the globe for the last half century. *Environmental Research Letters* **16**, 064006 (2021).
